# Survival and mortality in cerebral palsy: observations to the sixth decade from a data linkage study of a total population register and National Death Index

**DOI:** 10.1186/s12883-019-1343-1

**Published:** 2019-06-04

**Authors:** Eve Blair, Katherine Langdon, Sarah McIntyre, David Lawrence, Linda Watson

**Affiliations:** 1 Telethon Kids Institute, PO Box 855, West Perth, 6872 WA Australia; 2 University of Western Australia, Crawley, 6009 WA Australia; 3 Department of Paediatric Rehabilitation, Perth Children’s Hospital, Nedlands, 6009 WA Australia; 4 Cerebral Palsy Alliance, Sydney, New South Wales Australia; 5 University of Sydney, Sydney, New South Wales Australia; 6 Graduate School of Education, University of Western Australia, Crawley, Australia; 7 WA Register of Developmental Anomalies, King Edward Memorial Hospital, PO Box 134, Subiaco, 6904 WA Australia

**Keywords:** Cerebral palsy, Data linkage, Survival, Life expectancy, Time trends, Total population, Western Australia

## Abstract

**Background:**

Likely duration of survival of children described as having cerebral palsy is of considerable interest to individuals with cerebral palsy, their families, carers, health professionals, health economists and insurers. The aim of this paper is to describe patterns of survival and mortality to the sixth decade in a geographically defined population of people with cerebral palsy stratified according to the clinical description of their impairments in early childhood.

**Methods:**

Identifiers of persons born in Western Australia 1956–2011, registered with cerebral palsy on the Western Australian Register of Developmental Anomalies and surviving at least 12 months, were linked to the Australian National Death Index in December 2014. Patterns of mortality were investigated using survival analysis methods.

**Results:**

Of 3185 eligible persons, 436 (13.7%) had died. Of that sample the 22% with the mildest impairment had survival patterns similar to the general population. Mortality increased with increasing severity of impairment. Of 349 (75%) with available cause of death data, 58.6% were attributed to respiratory causes, including 171 (49%) to pneumonia at a mean age of 14.6 (sd 13.4) years of which 77 (45%) were attributed to aspiration.

For the most severely impaired, early childhood mortality increased in succeeding decades of birth cohorts from 1950s to 1990 with 20% dying by 4 years of age in the 1981–1990 birth cohort; it then decreased for subsequent birth cohorts, 20% mortality not being attained until 15 years of age. However by 20 years of age mortality of the most severely impaired born in the 1991–2000 birth cohort exceeded that of all other birth cohorts.

Remaining life expectancies by age to 50 years have been estimated for two strata with more severe impairments.

**Conclusion:**

For 22% of individuals with cerebral palsy with mild impairment survival to 58 years is similar to that of the general population. Since 1990 mortality for those with severe cerebral palsy in Western Australia has tended to shift from childhood to early adulthood.

## Background

Estimates of likely duration of survival with severe cerebral palsy (CP) continue to be of considerable interest to individuals with CP, their families, carers, health professionals, health economists and insurers. Longitudinal CP registers ascertaining all cases born within a defined geographical area form the ideal basis from which to conduct survival studies stratified by descriptions of early impairments. Many such registers now exist internationally, providing locally relevant survival statistics, however the majority are of quite recent origin and therefore can only report on somewhat limited durations of survival. Register longevity is dependent on continuous funding which can be difficult to source [1]. In this respect Western Australia is fortunate to have now secured, following community consultation, statutory notification of CP to the State held Western Australian Register of Developmental Anomalies. Service records provide another source of data from which survival studies may be conducted which are less vulnerable to funding problems but can seldom be considered population based unless they are the sole service provider for a geographically isolated population. The advantage of service records is that they are less limited by the longevity of the service since persons access the service at variable ages. The consequence of this is that the descriptions of their impairments are likely to be those at the age at which services are received. While CP is defined as a non-progressive condition, it is the cerebral anomalies that are non-progressive, the resulting physical impairments change over the lifespan particularly for the more severely impaired. The subject was reviewed in a 2006 publication which presented survival curves to 40 years of age [2].

The survival to June 1997 of people with CP born in Western Australia (WA) between 1956 and 1994 was described in our previous paper [3]. Mortality was highest in the first five years of life, severe/profound intellectual disability was the strongest single predictor of death followed by severe motor impairment and no significant improvement within groups defined by their disabilities in survival were observed over the study period despite advances in medical care.

More recent CP register studies from Western Sweden [4] and Victoria, Australia [5] made similar observations, but the interpretation of the analysis of a large CP service cohort in California (1983–2010) [6] was that ‘the trend towards improved survival has continued throughout the most recent decade’, though the improvements were not observed in all disability and age groups.

The objective of the current paper is to extend our original birth cohort by 16 years, adding births 1995–2011 and considering deaths occurring up to December 2014, thereby extending the period of observation for the earliest registrants well into the 6th decade. Survival is examined by severity of disability and across birth cohorts and compared with that of the general population.

## Methods

### Study participants and origin of data

What was formerly known as the WA Cerebral Palsy Register has been incorporated into the Western Australian Register of Developmental Anomalies (WARDA) and includes persons with CP born in WA from birth year 1956 onwards. Children meeting the criteria for CP acquired before 5 years of age have been actively ascertained from multiple sources since the inception of the register in 1977 [7]. Retrospective ascertainment among births prior to 1977 was exhaustively performed for a study of the incidence of CP in births 1956–75, examining records of all public and private service providers both in WA and in other Australian States as detailed by Stanley [8]. The medical records of children ascertained by the register prior to 5 years of age are followed until death or 5 years of age (whichever occurs first) and the description of impairments at the medical exam closest to 5 years is recorded on the register. The age of 5 years was chosen because by this age most impairments due to progressive syndromes (by definition excluded from CP) will have been identified, impairments that are going to resolve will have done so (and excluded) and the clinical picture is clearer since co-morbidities, particularly cognitive deficits, can be better ascertained in childhood than infancy. Unusually a person meeting all the register’s requirements for registration comes to the attention of the register more than 5 years after their birth. Their medical records are examined for details of their impairments recorded at the medical exam performed closest to 5 years of age, and these are added to the register. The register does not follow registrants after 5 years of age.

For these analyses, only those registrants surviving at least 12 months after birth were selected since many registers do not accept an incontrovertible description of CP prior to 12 months, nor would the sample be complete since infants who would have acquired the CP label had they survived will not be included. On account of this exclusion, remaining life expectancies are estimated from 1 year of age. Although the registered descriptions of impairments are those reported close to 5 years of age, the neurological damage responsible for those impairments existed before that age: at least since the perinatal period for those pre- and perinatally acquired and from the age of acquisition for the post neonatally acquired, which for half of whom was below 1 year of age [7].

Death information was sought in the (Australian) National Death Index (NDI) to identify all deaths of WA born CP registrants who had died within Australia. WARDA sent identifiers of all CP registrants born in WA 1956–2011 to the Australian Institute of Health and Welfare (AIHW), the custodians of the NDI. For every CP registrant with a possible match to the NDI, the AIHW added the date and any recorded cause(s) of death and these were verified by WARDA. Before submitting the file to the author, all personal identifiers were replaced with a study number (to de-identify participants while still allowing further enquiry should data cleaning necessitate this) and data describing the impairments and perinatal characteristics of each registrant were added.

### Overall disability score (DISAB)

Clinical descriptors of impairments when the participants reach 5 years of age that were used in these analyses are necessarily those that have been used since the inception of the WA CP register, since descriptors commonly used today (eg. the gross motor function classification system (GMFCS)) are not available for participants registered before these descriptors existed, which constitute the majority of our sample. Severity of motor impairment at the most impaired part of the body is categorised as minimal if abnormal neurological motor signs are present but confer little functional impairment, mild if there is some functional impairment, severe if there is little or no purposeful voluntary action possible and moderate if function lies between mild and severe.

The combined impact on survival of several co-existing impairments was investigated with the overall disability score (DISAB) described in our previous paper [3] (Table 1). DISAB can take values from 1 (minimal hemiplegia without additional impairment) to 12 (severe quadriplegia, bilateral blindness and deafness with active epilepsy and severe cognitive impairment). Intermediate scores are clinically more variable: e.g. a score of 6 could reflect severe total body motor impairment without additional impairment at one extreme or at the other a mild unilateral motor impairment with epilepsy and severe cognitive impairment.

**Table 1:** The overall disability score

DISAB (overall disability score) = Sum of	
extent of impairment (takes values 1 (*unilateral*), 2 (*bilateral predominantly lower limb*) or 3 (*bilateral other*))	
+ severity of impairment of the most impaired body part (0 (*minimal*), 1 (*mild*), 2 (*moderate*) or 3 (*severe*))	
+ cognitive impairment (0, 1 (*~IQ 50–69*), 2 (*~ 35–49*) or 3 (*< 35*))	
+ active epilepsy (0, 1)	
+ bilateral blindess (0, 1)	
+ bilateral deafness (0, 1)	

### Statistical methods

Frequency distributions were obtained of the characteristics of participants and causes of death. Mortality rates and standardised mortality ratios (SMR) were estimated by age (up to 58 years) and DISAB, with exact 95% confidence intervals of the SMRs obtained using the online calculator http://web1.sph.emory.edu/users/cdckms/exact-midP-SMR.html. Kaplan-Meier survival curves were plotted with *proc lifetest* (SAS version 9) by each participant characteristic including year of birth. The log rank test was used to estimate the reported significance of differences between survival curves. In each age group (1–4 years and thereafter in 5 year age bands) and in each disability score band (DISAB 1–5, 6–8 and > =9) mortality rate (obtained using *proc summary*, SAS version 9) was compared with that reported in Australian life tables 1995–97 [9]. Cox regression analyses estimated hazard ratios for those post neonatally acquired relative to pre-and perinatally acquired both with and without including DISAB in the model. The statistical significance of trends was estimated with Chi square or from regression analyses as appropriate.

### Estimating life expectancy

Life expectancy was calculated using the method of abridged life tables [10, 11]. Life expectancy is defined as the mean survival time for a group of individuals (i.e. the total number of years lived divided by the number of individuals in the group). Since most members of our study sample were alive at the study’s census date the total number of years lived is not available and life expectancy cannot be calculated directly from our data, assumptions concerning the likely mortality rates in people over age 60 years are required. These were estimated using the assumption of proportion life expectancy [12].

## Results

There were 3213 registrants on the WA CP register born in WA between 1956 and 2011. This data set was linked to the NDI in December 2014. Of these 3213 registrants 28 had died before 1 year of age. These analyses therefore consider 3185 CP registrants born in WA 1956–2011 and surviving at least 1 year, of whom 436 (13.7%) had died as of December 2014 and 2749 had not.

Their neonatal and impairment characteristics are shown in Table 2. The distributions of registrant characteristics have changed over the long study period. For example, the proportion born extremely preterm (< 28 weeks) has increased from < 1 to 10% (chi square for trend = 89.8 on 5 degrees of freedom, *p* < 0.0001) and median DISAB decreased from 6 to 4, (regression coefficient − 0.152 per decade (95% CI -0.218, − 0.082) *p* < 0.0001. Among the 95.7% with sufficient data to calculate a DISAB score, each of the scores 1 to 12 was represented, although only 13 cases (0.43%) had a score of 12, since bilateral deafness is an infrequent co-occurring impairment.

**Table 2:** Impairment and neonatal characteristics of the sample

Characteristic	Survivors (max 2749) %^a^ (n)	Deaths (max 436) %^a^ (n)
Primary CP type		
Spastic hemiplegia	37.3 (1021)	12.0 (52)
Spastic diplegia	36.6 (1001)	16.9 (73)
Spastic quadriplegia	10.3 (282)	53.1 (230)
Ataxia	7.7 (210)	2.8 (12)
Dyskinesia	7.1 (193)	12.9 (56)
Hypotonic CP	1.1 (31)	2.3 (10)
Datum missing %	0.4 (11)	0.7 (3)
Mixed spastic quadriplegia+dyskinetic	2.2 (60)	8.9 (17)
Estimate of cognitive ability		
Normal/Borderline	61.2 (1658)	11.1 (48)
Mild	18.0 (487)	17.4 (75)
Moderate	9.2 (248)	9.5 (41)
Severe/Profound	11.7 (318)	62.0 (268)
Datum missing%	1.4 (38)	0.9 (4)
Epilepsy	29.3 (784)	69.0 (294)
Datum missing%	2.6 (72)	2.3 (10)
Blind	6.8 (181)	30.3 (126)
Datum missing%	3.0 (84)	4.6 (20)
Deaf	3.0 (79)	9.1 (36)
Datum missing%	3.1 (99)	8.9 (39)
Decade of birth		
1956–60	4.4 (121)	9.6 (42)
1961–70	12.6 (347)	24.8 (108)
1971–80	14.5 (398)	17.2 (75)
1981–90	19.0 (522)	23.6 (103)
1991–2000	24.6 (677)	18.2 (79)
2001–2011	24.9 (684)	7.7 (29)
Male	56.5 (1552)	58.5 (255)
Birthweight		
> =2500 g	66.0 (1470)	75.5 (308)
1500-2499 g	19.5 (434)	19.1 (78)
< 1500 g	14.5 (324)	5.4 (22)
Datum missing %^2^	18.9 (521)	6.4 (28)
Gestation of birth		
> =37 weeks	66.3 (1732)	79.6 (328)
32–36 weeks	15.5 (405)	13.1 (54)
< 32 weeks	18.1 (473)	7.3 (30)
Datum missing %	5.1 (139)	5.5 (24)
Overall Disability score		
1–5	70.9 (1871)	14.4 (59)
6–8	17.3 (456)	21.5 (88)
9–12	11.8 (311)	64.1 (262)
Datum missing %	4.0 (111)	6.2 (27)

^a^ Proportion of those for whom that datum was not missing

Immediate causes of death by disability score and duration of survival are shown in Table 3. The mean DISAB score was lower for survivors (4.6 (sd 2.5), median 4) than for those who had died (8.5 (sd 2.6) median 9) although causes associated with aging in the general population (cancer, major organ failure, circulatory problems) were associated with a lower DISAB (median 6) and with median ages of death in the range 25–37 years, older than CP deaths from other causes but younger than would be expected in the total population.

**Table 3:** Distribution of causes of death with associated characteristics of overall disability score (DISAB) and duration of survival

Coded Cause of death	Total % (N)	Mean DISAB(sd)	Median DISAB	N with missing DISAB	Mean age of death years (sd)	Median age of death, years	Range of age of death, years
Respiratory	56.8 (187)	9.3 (1.9)	10	2	15.0 (12.6)	12.1	1.0, 55.6
No sufficient immediate cause coded	7.3 (24)	8.3 (3.0)	9.5	0	23.7 (16.8)	21.1	1.0, 50.8
Status epilepticus	6.7 (22)	7.4 (2.5)	7.5	0	9.4 (9.2)	6.1	1.3, 36.8
Accident/trauma	6.4 (21)	6.1 (2.4)	6	0	22.5 (14.1)	21.3	2.5, 54.6
Non-respiratory infection	4.9 (16)	8.5 (2.7)	10	1	16.6 (15.9)	11.5	1.3, 53.6
Cancer	4.0 (13)	6.1 (2.35)	6	0	37.7 (14.5)	36.6	18.9, 57.2
Circulatory disease	3.6 (12)	7.5 (2.4)	7	1	28.4 (17.9)	24.0	2.4, 55.1
Major organ failure	3.0 (10)	6.5 (2.8)	6	0	26.8 (16.5)	25.9	4.1, 49.9
> 1 sufficient cause	3.0 (10)	8.6 (2.9)	9	1	23.2 (6.3)	25.0	1.8, 51.2
Miscellaneous	2.7 (9)	9.6 (1.8)	10	0	15.5 (9.8)	14.0	5.5, 37.7
Birth defects	1.5 (5)	7.4 (3.6)	9	0	14.6 (18.2)	5.5	2.0, 45.3
Total with cause	100 (329)	8.6 (2.4)	9	5	17.8 (14.7)	14.3	1.0, 57.2
	N						
Unknown cause	107	8.1 (2.6)	9	85	11.6 (10.2)	7.9	1.0, 39.8
Total deaths	436	8.5 (2.5)	9	90	16.3 (14.0)	12.5	1.0, 57.2
					Mean age at census	Median age at census	Age range at census
Survivors	2749	4.6 (2.5)	4	111	25.6 (15.0)	23.4	2.1, 58.1

### Survival analysis

#### By overall disability score (DISAB)

**Fig. 1:**
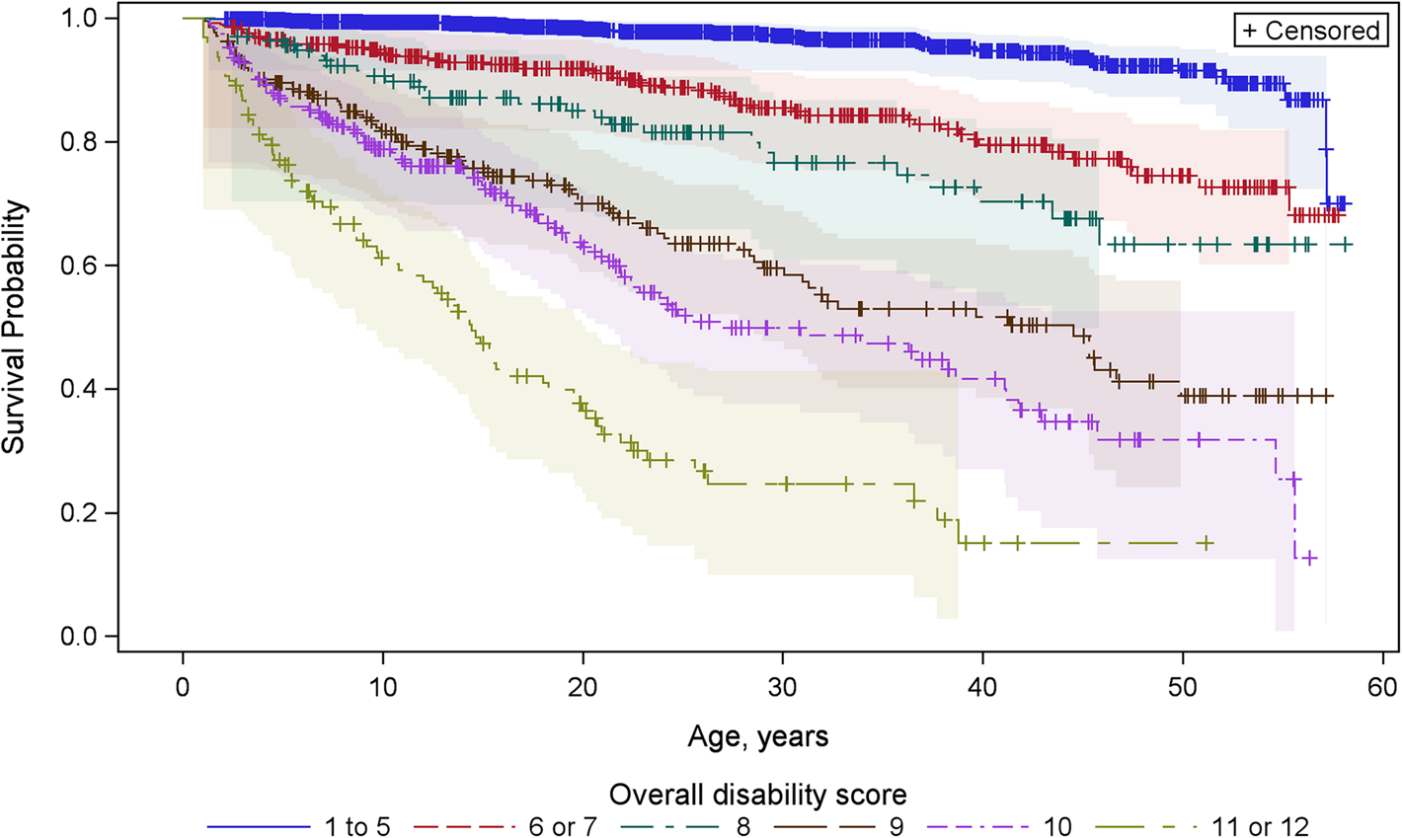
Survival curves by overall disability score (DISAB)

SMRs stratified by each of the 12 possible DISAB scores are shown in Table 4. Although SMR increased with increasing DISAB scores 1 through 5, the differences were not statistically significant on account of the low number of deaths among the most mildly affected, resulting in very wide 95% confidence intervals. Survival continued to decrease with increasing score, though survival with a score of 6 was similar to that with 7 and survival with a score of 11 similar to that with 12, suggesting six statistically significantly different disability groups with respect to mortality to 58 years (*p* < 0.0001). Survival curves stratified by these six groups (DISAB 1–5, 6–7, 8, 9, 10, 11–12) are shown in Fig. 1 which indicates 50% mortality by 15 years of age for those with DISAB of 11–12; by 22 years for those with DISAB of 10; by ~ 35 years with DISAB of 9 and more than 58 years for those with lower scores. On account of the limited numbers with higher DISAB scores, in subsequent analyses DISAB was stratified into three groups: DISAB scores 1–5, 6–8 and 9–12.

**Table 4:** Standardised mortality ratio (SMR) by disability score

Disability score	N	N deaths	N Expected deaths	SMR (exact 95% CI)	SMR (95% CI) of combined DISAB groups
1	123	1	1.432	0.698 (0.35, 3.44)	1.62 (1.24, 2.08)
2	463	5	6.651	0.752 (0.28, 1.67)	
3	558	18	10.53	1.709 (1.05, 2.65)	
4	488	18	9.590	1.877 (1.15, 2.91)	
5	302	15	6.939	2.162 (1.26, 3.49)	
6	235	32	5.225	6.124 (4.26, 8.54)	6.43 (4.93, 8.25)
7	176	26	3.790	6.861 (4.58, 9.91)	
8	138	31	2.673	11.60 (8.02, 16.3)	11.6 (8.02, 16.3)
9	225	92	3.344	27.51 (22.3, 33.6)	27.5 (22.3, 33.6)
10	242	106	2.574	41.19 (33.9, 49.6)	41.2 (33.9, 49.6)
11	117	74	0.677	109.3 (86.5, 136.5)	110 (88.7, 136)
12	13	9	0.077	116.7 (56.9, 214.2)

The proportion of the CP population with DISAB scores 9–12 remained constant at just below 20%, but the proportion with scores 1–5 increased from one half in the 1950s to two thirds in the new millennium, balanced by a decrease in the proportion with DISAB scores 6–8 (Chi square test for trend =44.5 on 10 degrees of freedom, *p* < 0.0001). At the same time DISAB scores tended to increase in the DISAB> = 9 stratum (T-test of linear regression gave a regression co-efficient of + 0.06 (95% CI 0.02, 0.11) *p* = 0.005) and decrease in the DISAB <=5 stratum regression coefficient = − 0.14 (95% CI -0.17, − 0.11) p < 0.0001 with no systematic change in DISAB distribution over birth cohorts in the DISAB 6–8 stratum (*p* = 0.88).

#### By year of birth

Year of birth was stratified by decade: the first stratum covered the 5 years 1956–1960, subsequent strata each covered 10 years (eg. 1961–1970) until the sixth stratum which encompassed 11 years, 2001–2011.

**Fig. 2:**
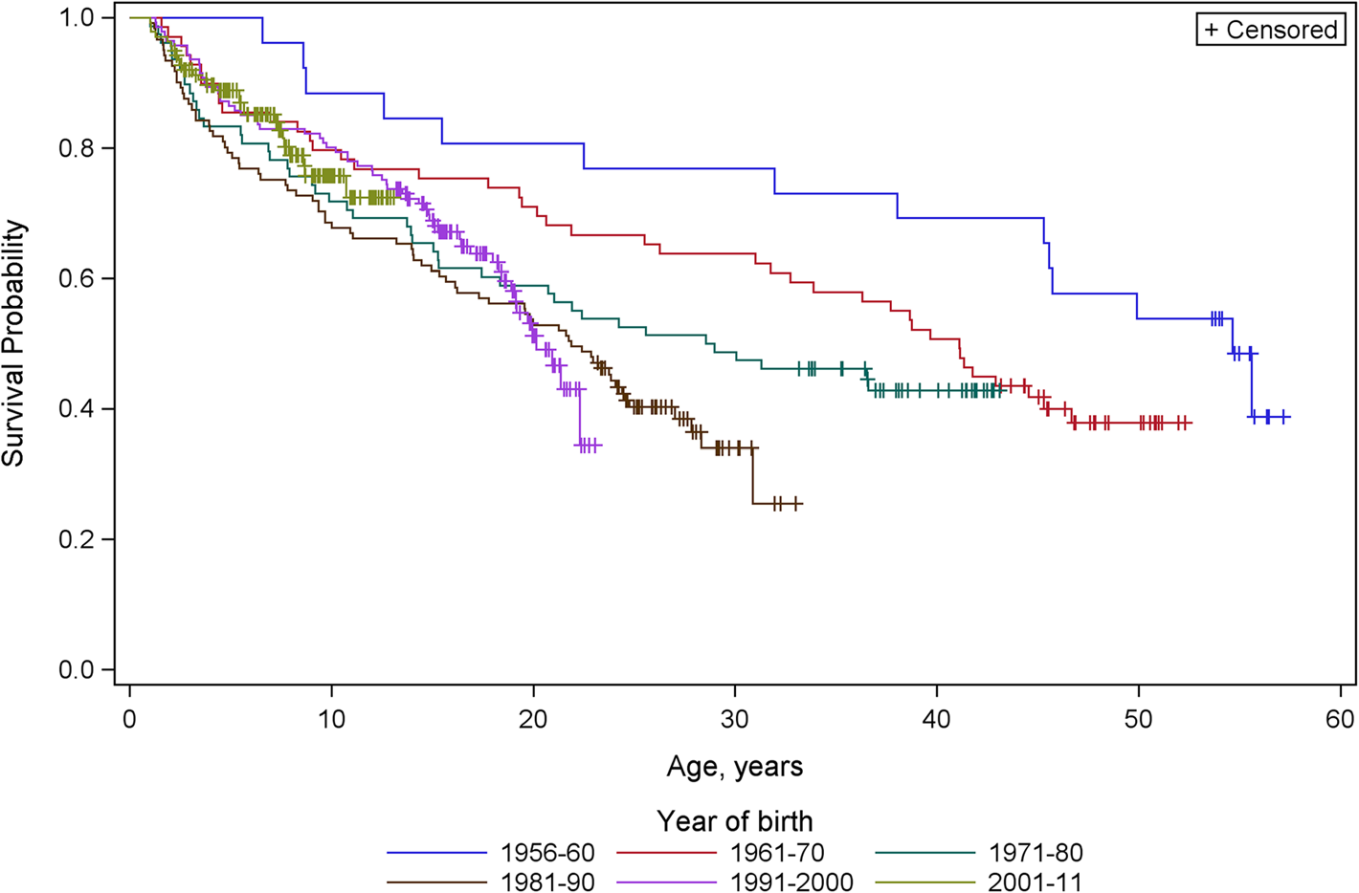
Survival curves for those with DISAB > = 9 by decade of birth

Survival analysis by decade of birth of those with DISAB > = 9 is shown in Fig. 2 in which differences between the curves, estimated by log-rank test, are statistically highly significant (*p* < 0.0004). Examination of the curves suggests the following: that registrants born in the first four year-of-birth strata (1956–1990) showed somewhat similar survival patterns: mortality rate was highest in early childhood and decreased with age until middle-age, but the early childhood rate increased in each subsequent decade. The two most recent birth cohorts (1990s and 2000’s) show different survival patterns: mortality rate in the first decade of life was lower than in the 1970s and 80s but did not decrease with age. In contrast to the first three strata, the curves suggest that those born in the 1990s experienced accelerating mortality in early adulthood such their survival to 20 years was the lowest of any year of birth decade. Births in the 2000s were too young at the census date to indicate whether this profile of accelerating mortality in early adulthood will be repeated, but their profile of mortality in early childhood overlays that of the 1990s cohort, with the suggestion of higher mortality on attaining school age. The 1980s birth cohort showed a transitional pattern: while mortality was highest early childhood (as in previous cohorts) it did not continue to decrease with age through adolescence and adulthood.

**Fig. 3:**
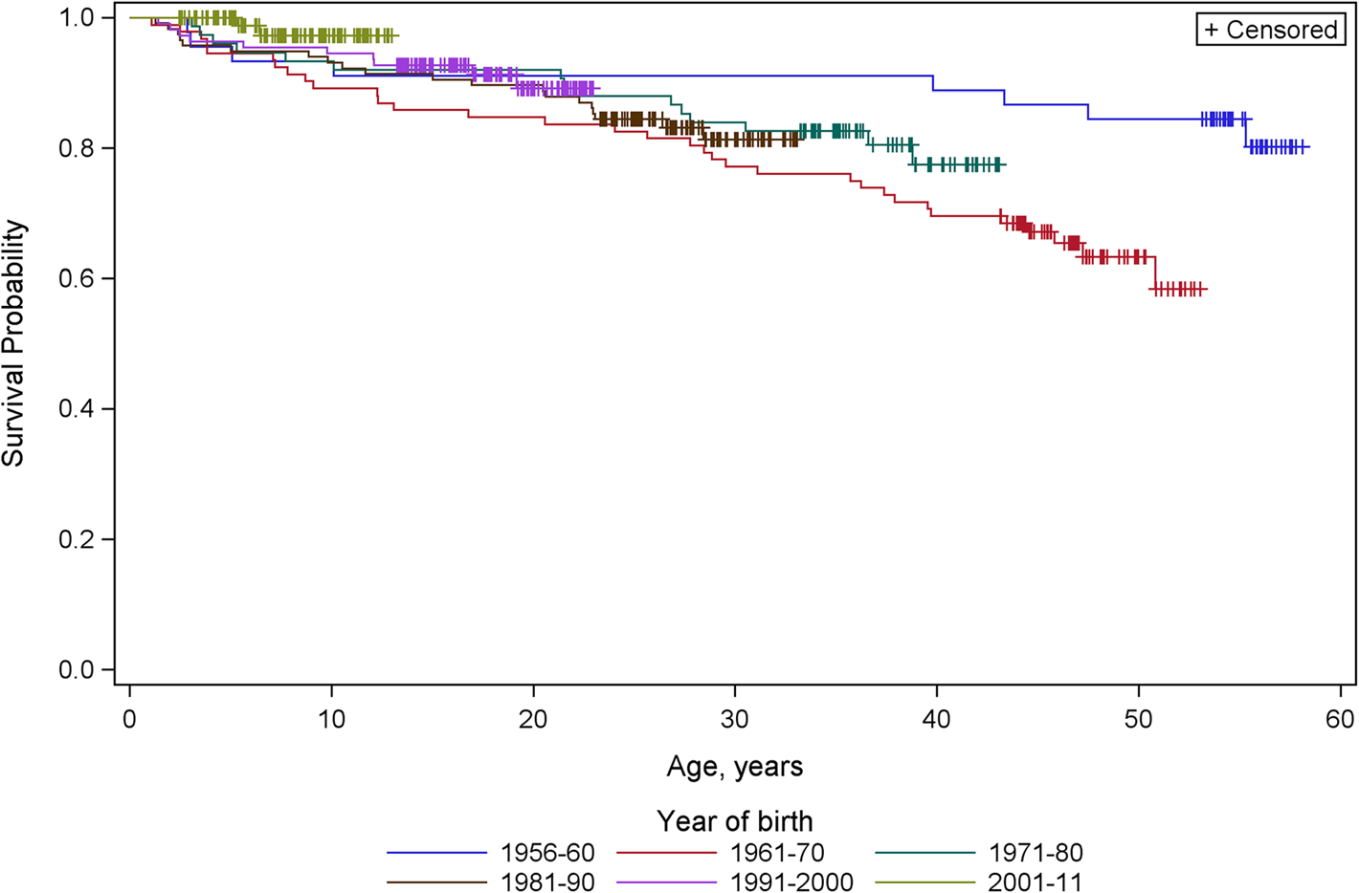
Survival curves for those with DISAB of 6, 7 or 8 by decade of birth

Survival analysis of those with DISAB score of 6, 7 or 8 showed much lower mortality rates that changed little with age. Their differences in survival patterns between the birth cohorts were estimated by log-rank test to be of marginal statistical significance (*p* = 0.049) with mortality being highest for the 1960s birth cohort and lowest (to 14 years of age) for the most recent cohort, Fig. 3.

#### By type of motor impairment

Among those with a DISAB score > =9, there were 389 with spastic CP and 186 with dyskinetic CP with or without additional spasticity. Median DISAB in both groups was 10 with similar mean DISAB of 9.94 for those with spastic CP and 9.74 for those with dyskinetic CP. Those with spastic CP showed greater early mortality which gradually decreased to the middle of the sixth decade, whereas those with dyskinetic CP experienced lower early mortality that increased in late adolescence/early adulthood with no further mortality after the mid-30s.

The numbers of registrants with a disability score > 5 and primarily ataxic or hypotonic CP were small: primary ataxia (*n* = 97) and primary hypotonia (*n* = 31). Mortality in those with hypotonic CP greatly exceeded that of those primarily ataxic, primarily because 11/31 with hypotonic CP had a DISAB score > =10, compared with only 2/97 with primarily ataxic CP. Limiting DISAB to the range 6–9 however still indicated significantly higher mortality (*p* < 0.0002) in those with hypotonic (60% survival to age 20 years) than with primarily ataxic CP (95% survival to age 20).

#### By time of acquisition of CP

Timing of brain damage was available for 3067/3185 (96.3%) of the sample. For 2700 (88.0%) it was considered pre- or peri-natal, for 367 (12.0%) a recognised post-neonatal cause occurred before the age of 5 years. Those with post neonatal acquisition had a statistically significantly shorter survival (*p* < 0.004). However, those with a recognised post-neonatal cause tended to have more severe impairment: 54.5% (*N* = 145) of post-neonatally acquired CP had a DISAB< 6 compared with 64% of pre-and perinatally acquired, and 27.1% (*n* = 72) had DISAB> = 9 compared with 18.4%. Controlling for DISAB in multivariable Cox regression reduced the statistical significance of post neonatal acquisition to *p* = 0.228, indicating that their greater degree of impairment was primarily responsible for the shorter survival of those with post-neonatally acquired CP.

#### Gender

In contrast to the general population, gender was not associated with mortality in those with CP, *p* = 0.877.

### Life expectancy

Estimated remaining life expectancies by age for those with DISAB > = 9 and those with DISAB 6–8 are shown in Table 5. As an example of how to read this table: if a person with DISAB> = 9 survives to 15 years of age, their remaining life expectancy is estimated as 31.9 years, so on average they could be expected to survive to 46.9 years of age: however should they continue to survive to 25 years of age, their remaining life expectancy is estimated to be 30.4 years, suggesting survival to 55.4 years of age.

**Table 5:** Estimated remaining life expectancy in years by age and overall disability stratum

Lower limit of age stratum, years	Overall disability score > =9	Overall disability score 6–8
	Remaining life expectancy, years	Remaining life expectancy, years
1	33.2	59.3
5	34.6	57.5
15	31.9	50.5
25	30.4	42.7
35	24.2	34.9
50	16.2	24.0

### Cause of death

Information concerning cause of death was available for 349 of the 436 (75.2%) deaths. Availability of cause of death data was associated with the year of death, being available for only 2 of the 42 (5%) cases dying before 1971, 48% of those dying in the 1970s increasing to 95% of those dying after 2000. Whether a cause of death was recorded was not associated with the severity of impairments.

The distribution of immediate cause of death is shown in Table 3. Death was attributed to respiratory problems for 56.8% (*n* = 187) of those with data concerning cause, principally pneumonia (82%) of which almost half (45%) were attributed to aspiration. A further 6% of respiratory deaths were attributed to choking. In addition, 5 of the 10 persons tabulated as having > 1 sufficient immediate causes of death died with complicated pneumonia.

The next most numerous category accounting for 6.9% (*n* = 24) did not have a satisfactorily explanatory cause. The coded ‘causes’ for this group included ‘cerebral palsy’, ‘respiratory arrest’, ‘cardiac arrest’, ‘found dead’ and even ‘senescence’. Although variable, mean DISAB was high (8.3) with a higher median score (9.5) indicating a positively skewed distribution which suggests that persons in this category may simply have been medically fragile. All 8 dying in childhood had DISAB scores > = 9, while the 5 with DISAB< 6 died in adulthood. This category was used more often in more recent deaths, accounting for 10.7% of deaths since the year 2000.

Causes of traumatic/accidental deaths (*n* = 21) were variable. Drowning, the most frequent cause (*n* = 5), was seen primarily in children. Other causes tended to be associated with deaths in adults with relatively low disability scores and may include suicides.

The miscellaneous causes include gastrointestinal and metabolic problems, combined to preserve anonymity.

For most causes the median age at death was several years below the mean age at death, indicating negatively skewed distributions. The exceptions were deaths attributed to cancer, major organ failure and circulatory problems other than cerebrovascular accidents, causes associated with aging in the general population which, as anticipated, were seen primarily in the earliest birth cohorts and more recent deaths.

Of the 10 persons tabulated as having > 1 sufficient immediate causes of death, the 6 with causes including cancer or major organ failure survived for more than 20 years, the other 4 died by age 5 years, with causes including complicated pneumonia. It should be noted that although only 10 persons were coded with > 1 sufficient immediate causes of death, many more had several medical problems in addition to either CP or the factor selected as the most likely immediate cause of death.

## Discussion

The long period of registration with consistent methods gives an indication of survival rates into the 6th decade by clinical observations made at 5 years of age which is available from only one other CP register internationally.

### Time trends

The increasing rate of early childhood mortality in those with DISAB > = 9 from one decade to the next for births from the 1950s to 1980s (Fig. 2) may be the result of the survival to their first birthday (and hence of inclusion in our sample) of increasingly compromised infants as a result of progress in obstetric and neonatal care. This is supported by our observation that mean DISAB score in the group with DISAB > = 9 tended to increase in succeeding birth cohorts.

The data concerning birth cohorts in the 1990s and 2000s therefore reflects a more complete sample of children with the cerebral anomalies that result in a description of CP including those with more severe functional impairments and marked medical vulnerability many of whom would previously have died perinatally or in infancy. Continuing progress and experience in neonatal and paediatric care for very compromised children is most likely responsible for the increased early survival in the 1990s and 2000s births cohorts, but this reduction in mortality was not sustained into later childhood and early adulthood. With a greater number of severely and multiply impaired children surviving to an age at which transition to adult services becomes mandatory (16 years in WA), adult services are now finding themselves in a position analogous to that of neonatal services some decades ago: they are being presented with problems with which they have very little prior experience. Neonatal services were presented with increasingly premature live births and with increasingly compromised more mature newborns. Initially survival of these infants was very poor but with increasing experience first survival improved, which was a pre-requisite, of course, to subsequent improvements in neurological outcomes in these very vulnerable patients. It has taken time to develop the expertise to successfully manage novel medical problems. Additionally, current adult medical services in Australia tend to be highly specialised and maybe ill equipped to deal with permanently disabled young people with multiple health problems who are best served by a wholistic approach to medical care and may require the advocacy of a third party. The increasing duration of survival of children with severe and multiple impairments brings with it the need to address the problems of their transition to adult services that are equipped to address their needs, yet those needs are not necessarily well understood, see below under ‘Causes of death’.

It is difficult to directly compare our results with published studies of mortality in CP due to differences in sample selection, in methods of categorising their impairments, in analytical methods, statistics reported and the birth cohorts considered. Furthermore if, as we anticipate, duration of survival with severe and multiple impairments is dependent not only on obstetric and neonatal expertise but also on the policies governing application of that expertise (which jointly determine who survives the perinatal period to have the potential of acquiring the CP label) as well as social factors determining the care they receive throughout the lifespan, it would be surprising if mortality rates, even if estimated using the same methods, did not vary temporally and geographically. The main effect of such factors may be anticipated to be via the distribution of types and severity of impairments observed. For example in a comparison of studies from California, UK and WA, Shavelle, Strauss and Day showed that groups of children from each study with very similar levels of motor and cognitive impairments had very similar survival curves [13]. But determinants of survival other than the nature of impairments may exist. For example: another Australian register-based study published Kaplan Meier curves for children with severe motor impairment and at least 3 additional impairments, which we assume to be a sample similar to those with DISAB> = 9 in our study. As in our current study, they also found mortality to be higher in the 1980s birth cohort than the 1970s birth cohort, but their survival curve for the 1990s birth cohort overlay that of the 1980s, while ours showed less early childhood mortality but increasing rates of adolescent and young adult mortality: so although mortality rates were similar, the time trends did not appear to be [14].

### Causes of death

All studies of CP mortality investigating cause attribute more than half of observed deaths to respiratory problems but the exact nature of those problems, which may be specific to CP, is not well defined. Most such studies mention aspiration associated with oropharyngeal dysfunction and resulting pneumonia as important factors. e.g. [4]. These were also identified in a recent study of risk factors predicting respiratory hospital admissions in CP which also added seizures, gastro-oesophageal reflux disease and previous respiratory symptoms particularly those necessitating hospitalisation or antibiotic use, but not scoliosis [15]. These factors suggest that it is *chronic* lung disease which predisposes its sufferers to the recurrent pneumonia and respiratory failure seen in people with CP with oropharyngeal dysfunction. At the end of life it presents as an extremely marked susceptibility to respiratory infections during which repeated prolonged hospitalisations for respiratory support with oxygen or non-invasive ventilation may be required [16]. This may occur for some in childhood but our data suggest that this is increasingly delayed until adolescence or young adulthood and may coincide with the transition into adult care when care may be compromised on account of less ready access to familiar treating physicians [17].

The increased use of gastrostomies has offered better nutrition and limited aspiration primarily to saliva only, rather than food, drink and saliva. However gastrostomy does not prevent repeated multi-day hospital admissions [16] and it is likely that it delays rather than altogether prevents associated lung damage. Gut dysfunction and deterioration in foregut motility associated with autonomic dysfunction may contribute to the development of chronic respiratory disease by exacerbating gastro-oesophageal reflux which can cause vomiting and aspiration, regardless of the presence of a gastrostomy [18].

There is limited evidence concerning either the prevention or treatment of oropharyngeal dysfunction and chronic respiratory disease and their presence is neither well recognised nor managed at any time in the life of a person with CP [19]. They are often lost among the myriad of associated impairments and musculoskeletal and other complications of CP. The significance of respiratory disease associated with CP is only now gaining recognition because of its over representation as a cause of death in all age groups.

This study also found that people with CP who died of causes associated with aging in the general population such as cancer, major organ failure and circulatory problems, died at younger ages than would be seen in the total population and tended to have relatively low DISAB scores (median 6). This is consistent with the excess mortality due to ischaemic heart disease and cancer reported in adults with CP [20] and with a US population-based study showing that adults with CP have significantly higher odds of a range of chronic diseases compared with adults without CP [21]. This raises further questions about the management of preventable health complications in the CP population [19, 20], which may require adjustment of care throughout the lifespan.

### Limitations

Inevitably this study has limitations. Deaths among any registrants who have migrated overseas would not be identified by our methods, but such migration is considered rare for Australians with significant disability. Deaths among registrants before the age of 5 years risks misclassification both of the CP status and of their DISAB score. It is possible that some may have had an unrecognised progressive disease that would not have been included as CP had they survived to recognition of the progressive nature of their disease, though this is considered very unlikely since such cases would be strenuously investigated. Those that die before the age of 5 years tend to be very severely impaired: the more severe the impairments the earlier they can be recognised reliably. It is in the milder and moderately severely impaired persons that the degree of impairment is harder to judge in infancy, and these are most likely to survive beyond 5 years. Thus misclassification of the degree of impairment of early deaths is unlikely, particularly with the stratification of DISAB.

As discussed in Methods, many of the instruments now used to assess motor function in persons with CP have only become available in the last ten years. Here we are reporting on study subjects for sixty years, so for consistency we must continue with the methodology available for all subjects.

The concept of an overall disability score is valuable in predicting mortality since it considers factors not encapsulated by the GMFCS, now the most commonly used single indicator of severity of impairment. While obviously correlated it is not possible to draw one to one correlations between GMFCS and DISAB. Although the current DISAB is a composite score reflecting level of several aspects of functioning, it does not capture medical vulnerability such as that associated with very severe motor, particularly oropharyngeal, dysfunction, chronic lung disease or autonomic dysregulation. Thus as a predictor of mortality DISAB requires refinement, consideration of oropharyngeal dysfunction is essential since this is far more strongly associated with mortality than is, for example, deafness and a more rigorous assessment of cognitive ability is highly desirable, but the required measures were not available for much of this study’s sample.

## Conclusions

For 22% of our CP population with the mildest impairments, duration of survival to 58 years does not differ significantly from that of the population but, as anticipated, the SMR increases with increasingly severe impairments. Nonetheless more than 80% have a life expectancy beyond 58 years and longer follow up of population based samples with detailed descriptions of impairments is recommended to obtain a more complete picture of survival with CP. For those with severe and multiple impairments early childhood mortality has tended to decrease for births since 1990 and the majority reach adulthood.

## Data Availability

The original data sets are the property of Western Australian Register of Developmental Anomalies – Cerebral Palsy (WARDA-CP) and of the Australian Institute of Health and Welfare (National Death Index) respectively. The linked data set is to be retained by WARDA-CP for some years. These data are available from the custodial institution subject to appropriate approvals.

## Abbreviations

AIHW: Australian Institute of Health and Welfare
CP: Cerebral Palsy
DISAB: Overall disability score
GMFCS: Gross motor function classification system
MR: Mortality ratio
NDI: National Death Index *(of Australia)*
SMR: Standardised mortality ratio
WA: Western Australia
WARDA: Western Australian Registry of Developmental Anomalies
